# Supplementary material for: Rational Zika vaccine design via the modulation of antigen membrane anchors in chimpanzee adenoviral vectors
Source: Nat Commun. 2018 Jun 22;9:2441. doi: 10.1038/s41467-018-04859-5 (PMC6015009; doi:10.1038/s41467-018-04859-5)
Supplement: Supplementary file 1 — Supplementary Information [file 41467_2018_4859_MOESM1_ESM.pdf]

Supplementary information for:

Rational Zika vaccine design via the modulation of antigen membrane anchors in chimpanzee adenoviral vectors

Lopez-Camacho et al.

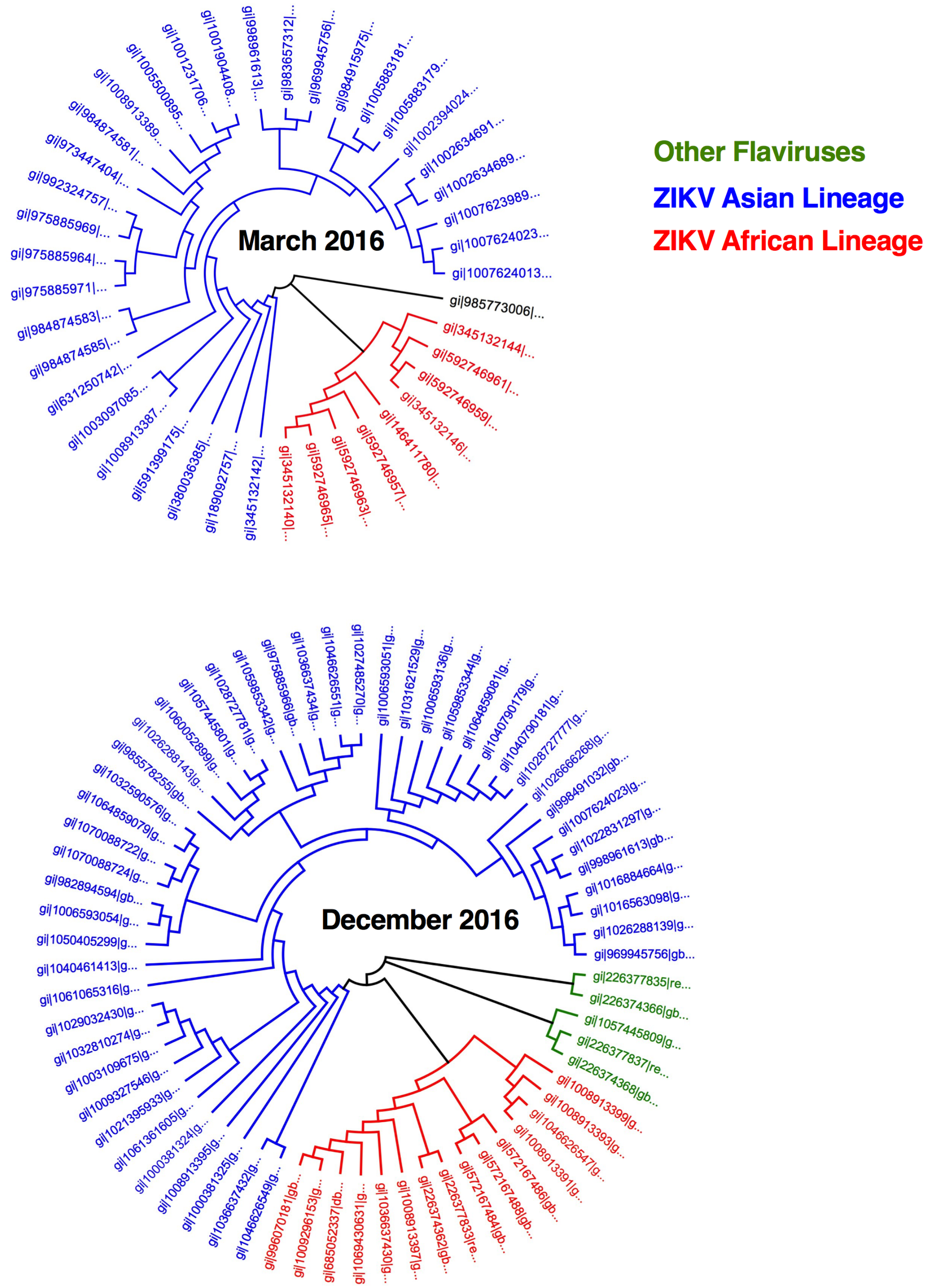

Supplementary Figure 1. ZIKV Phylogeny trees. Trees were constructed from sequences available in March and December 2016, using both Asian and African ZIKV full genomes. Other flavivirus are included as an example to show a hierarchical branching in the trees.

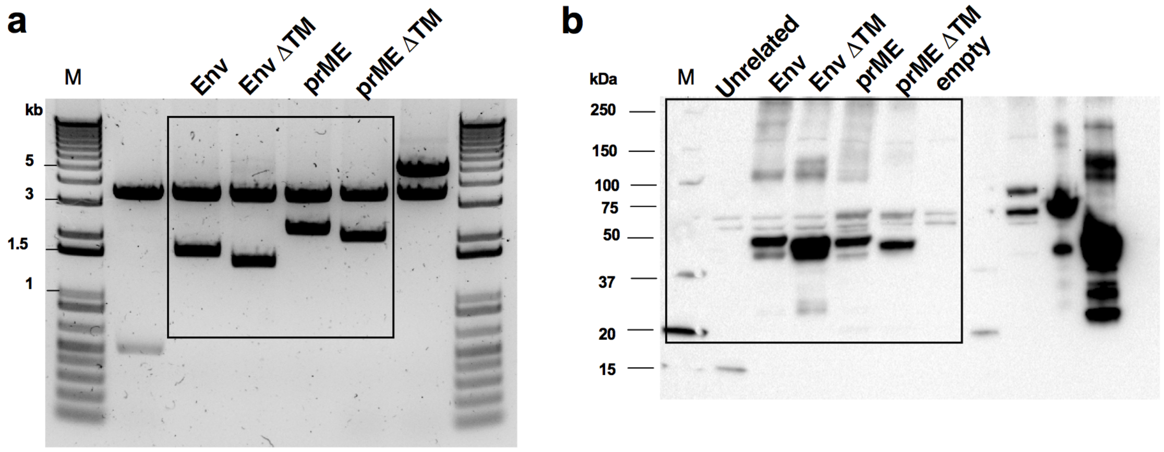

Supplementary Figure 2. Uncropped images from main Figure 1e and Figure 1d.

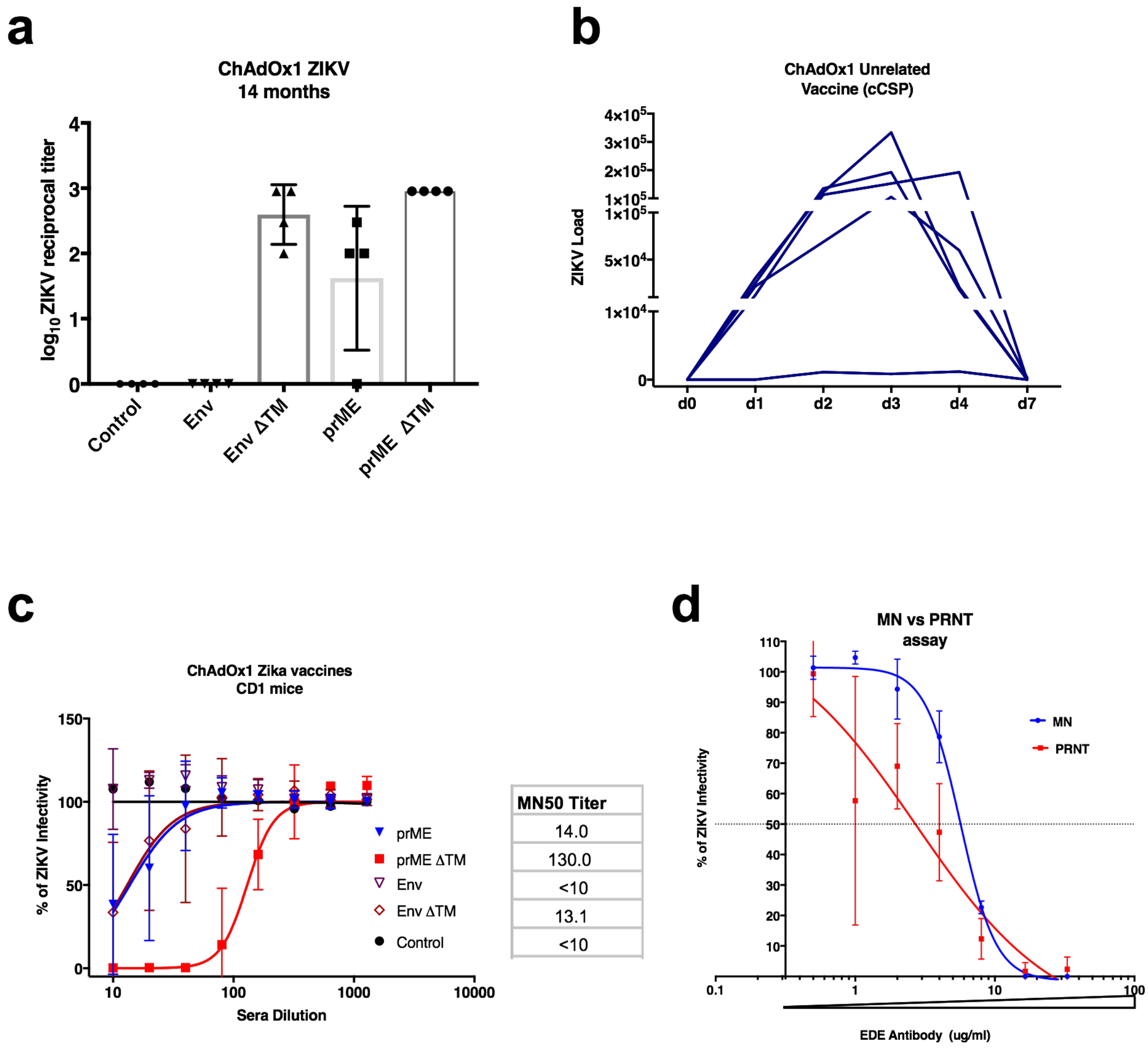

Supplementary Figure 3. Humoral Immunogenicity and ZIKV neutralisation assay of ChAdOx1 ZIKV vaccines. **(a)** Anti-envelope antibody responses elicited by Asian-based ChAdOx1 ZIKV vaccines in outbred CD1 mice, 14 months after a single immunisation. **(b)** BALB/c mice (n=5) immunised with a single i.m. shot of a ChAdOx1 unrelated vector were intravenously challenged with 10^5 VP of ZIKA-BR at week four after prime. **(c)** Neutralisation capacity of sera from vaccinated CD1 mice (Prime, 16 weeks), the graph depicts the ZIKV infectivity (100% = ZIKV infectivity in control/naïve sera). MN50 titers were calculated as the 50% reduction of ZIKV infectivity. Lines represent the nonlinear fit of a dose-response graph. Error bars are the SD of the mean. **(d)** Percentage of ZIKV infectivity comparison between the microneutralisation and traditional Plaque Reduction Neutralisation Assay (PRNT) in Vero cells. A highly neutralising monoclonal antibody EDE1 C8 was used at different concentrations. Lines represent the nonlinear fit of a dose-response graph. Error bars are the SD of the mean of three sample replicates.

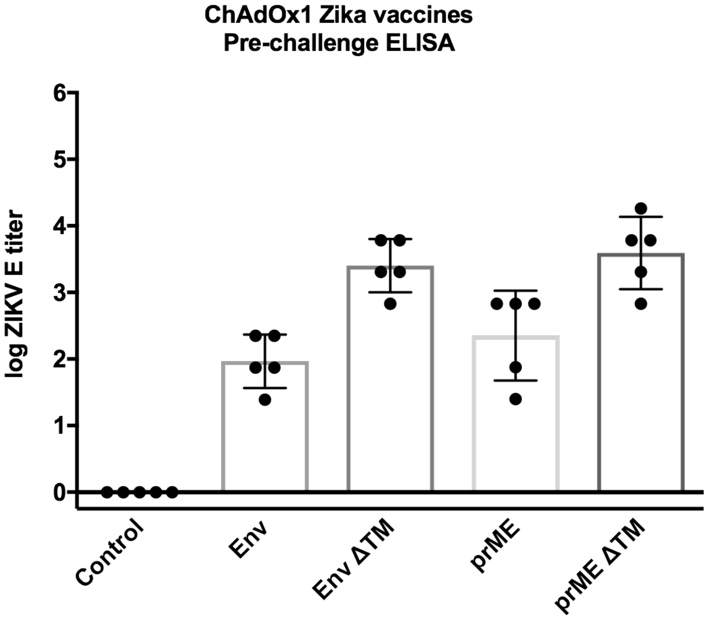

Tukey's multiple comparisons test	Mean Diff.	95.00% CI of diff.	Significant?	Summary	Adjusted P Value	
prME vs. prME ΔTM	-1.241	-2.177 to -0.3038	Yes	**	0.0079	A-B
prME vs. Env ΔTM	-1.05	-1.986 to -0.113	Yes	*	0.0255	A-C
prME vs. Env	0.3862	-0.5505 to 1.323	No	ns	0.6477	A-D
prME ΔTM vs. Env ΔTM	0.1908	-0.7458 to 1.128	No	ns	0.9358	B-C
prME ΔTM vs. Env	1.627	0.69 to 2.563	Yes	***	0.0007	B-D
Env ΔTM vs. Env	1.436	0.4992 to 2.373	Yes	**	0.0023	C-D

Supplementary Figure 4. Immunogenicity of ChAdOx1 ZIKV vaccines in pre-challenged sera. Reciprocal anti-envelope antibody titre was calculated for sera from all vaccinated groups by ELISA. Bars represent the mean of antibody titer, error bars are the s.d. and dots represent a single sera sample for each group. Table shows the statistical analysis (One-way ANOVA, Multiple comparison between vaccinated groups) with P values.

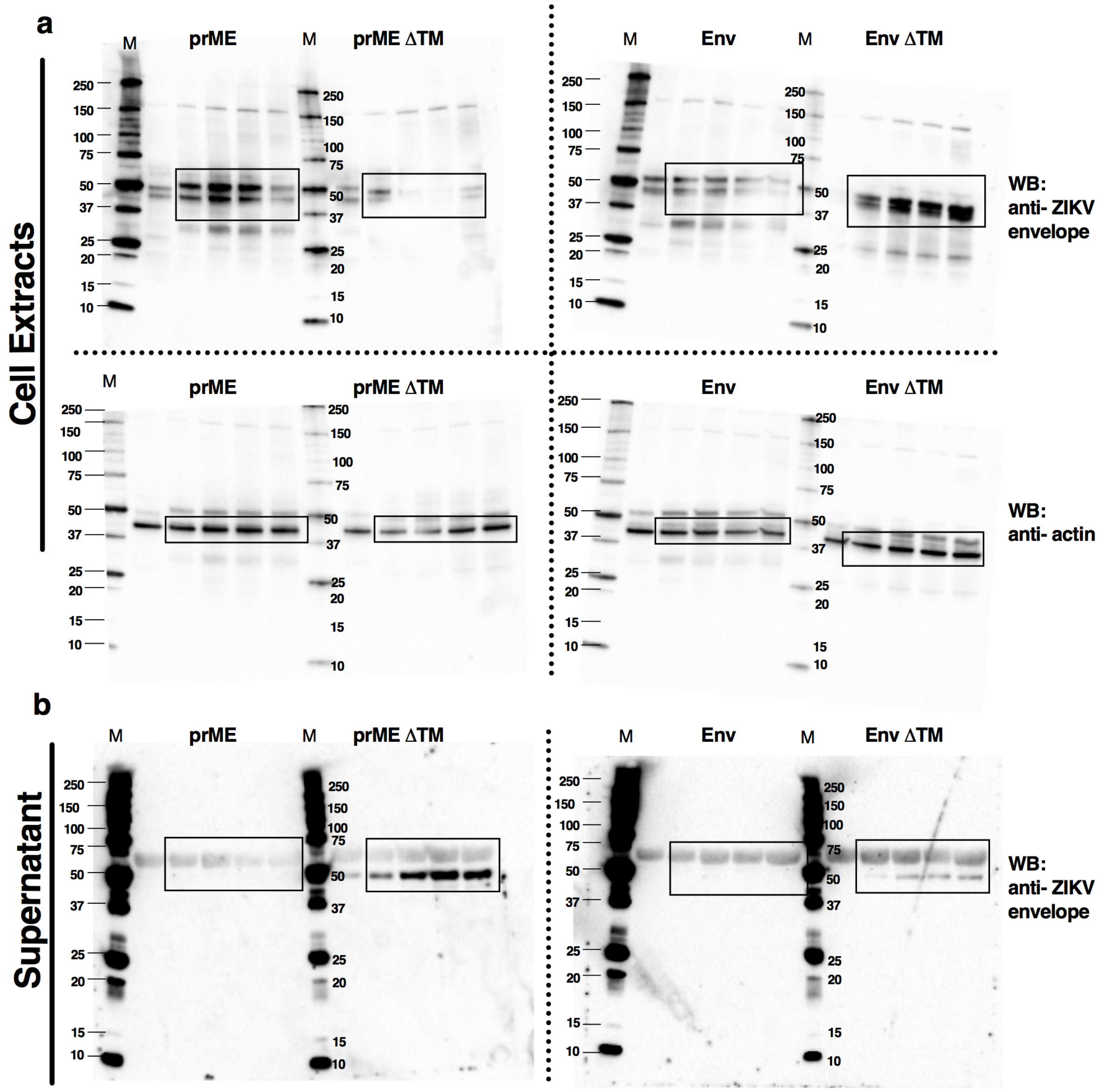

Supplementary Figure 5. Uncropped images from main Figure 3g.

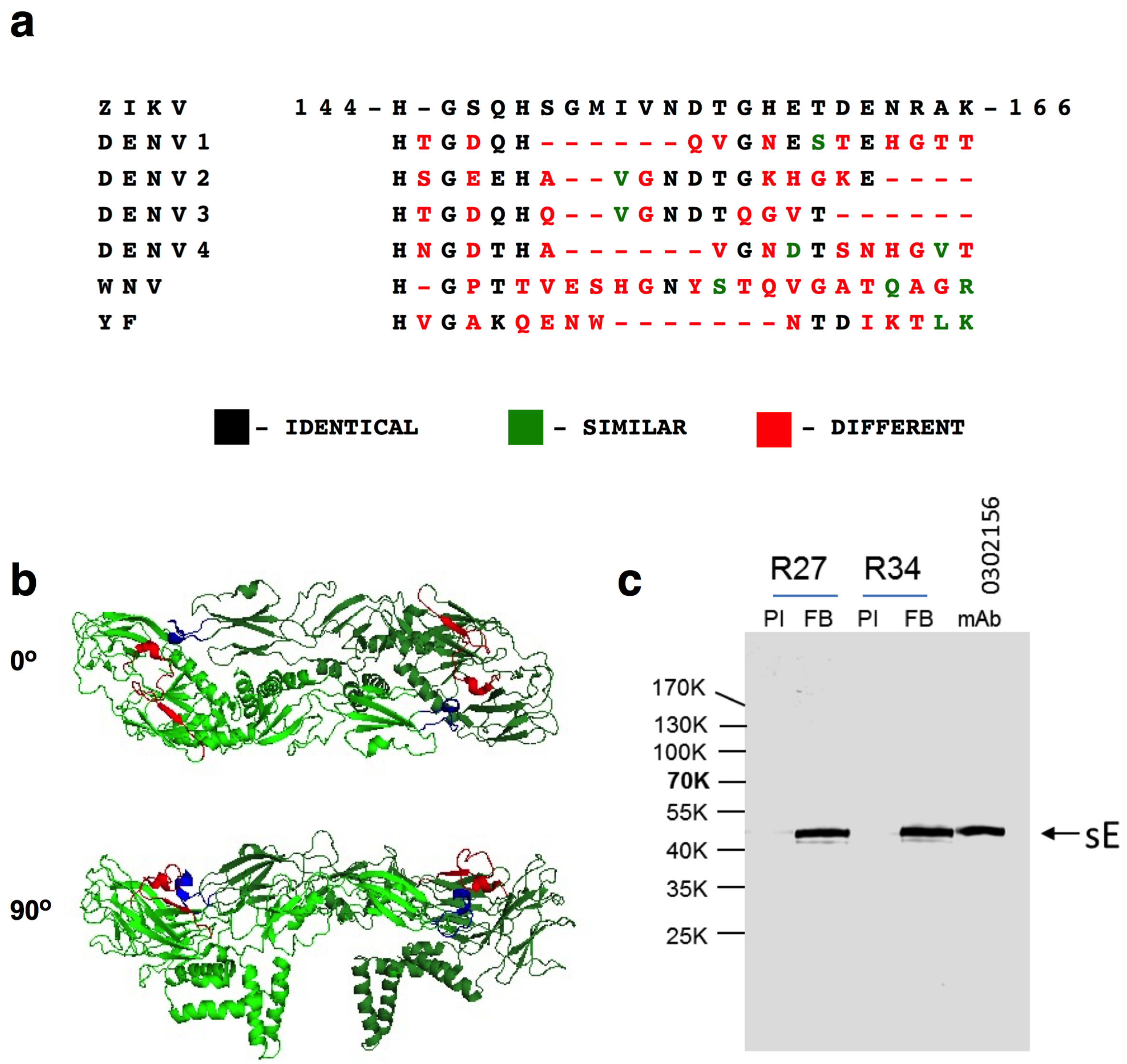

Supplementary Figure 6. Development of an antibody for the region 144 to 166 a.a. of ZIKV envelope. **(a)** ZIKV envelope protein sequence (Q91KX7) was aligned with DENV1 (Q7TGE4), DENV2 (AHB63923), DENV3 (ABA25838), DENV4 (Q80L18), WNV (Q91R00) and YFV (Q89286) envelope proteins using the Basic Local Alignment Search Tool (BLAST). **(b)** 3D representations of the ZIKV envelope dimer showing region 144-166 in red and the fusion loop (recognised by the antibody mAb D1-4G2-4-15) in blue. **(c)** Two rabbits (R27 and R34) were immunised and their sera (final bleed or FB) tested by Western blot for binding to recombinant baculovirus-expressed soluble ZIKV envelope protein (sE), i.e. ZIKV envelope lacking the C-terminal transmembrane domain. As expected, the sera taken from animals prior to immunisation (pre-immune (PI)) do not recognise envelope . mAb 0302156 is a commercial mAb to ZIKV envelope, used as a positive control.

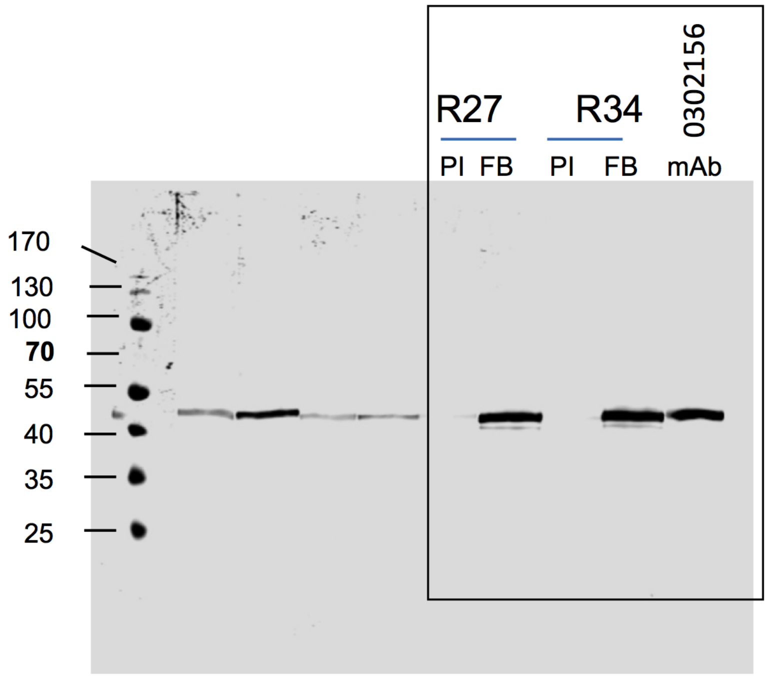

Supplementary Figure 7. Uncropped image from supplementary Figure 6c.

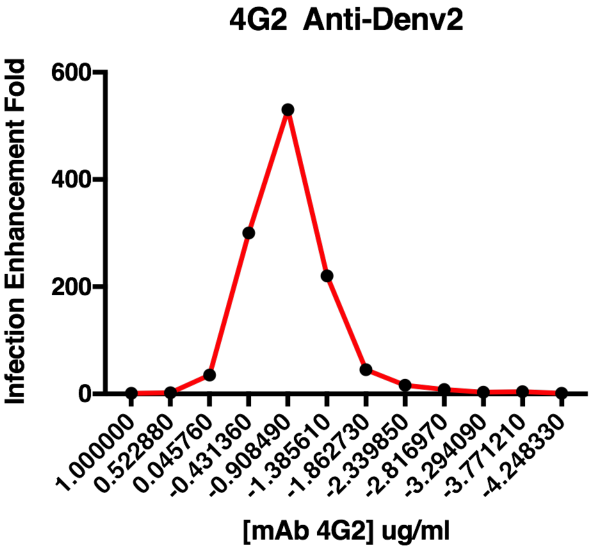

Supplementary Figure 8.

Infection of U937 cells by DENV2 in the presence of several antibody dilutions of a highly potent monoclonal neutralising DENV2 antibody (4G2).

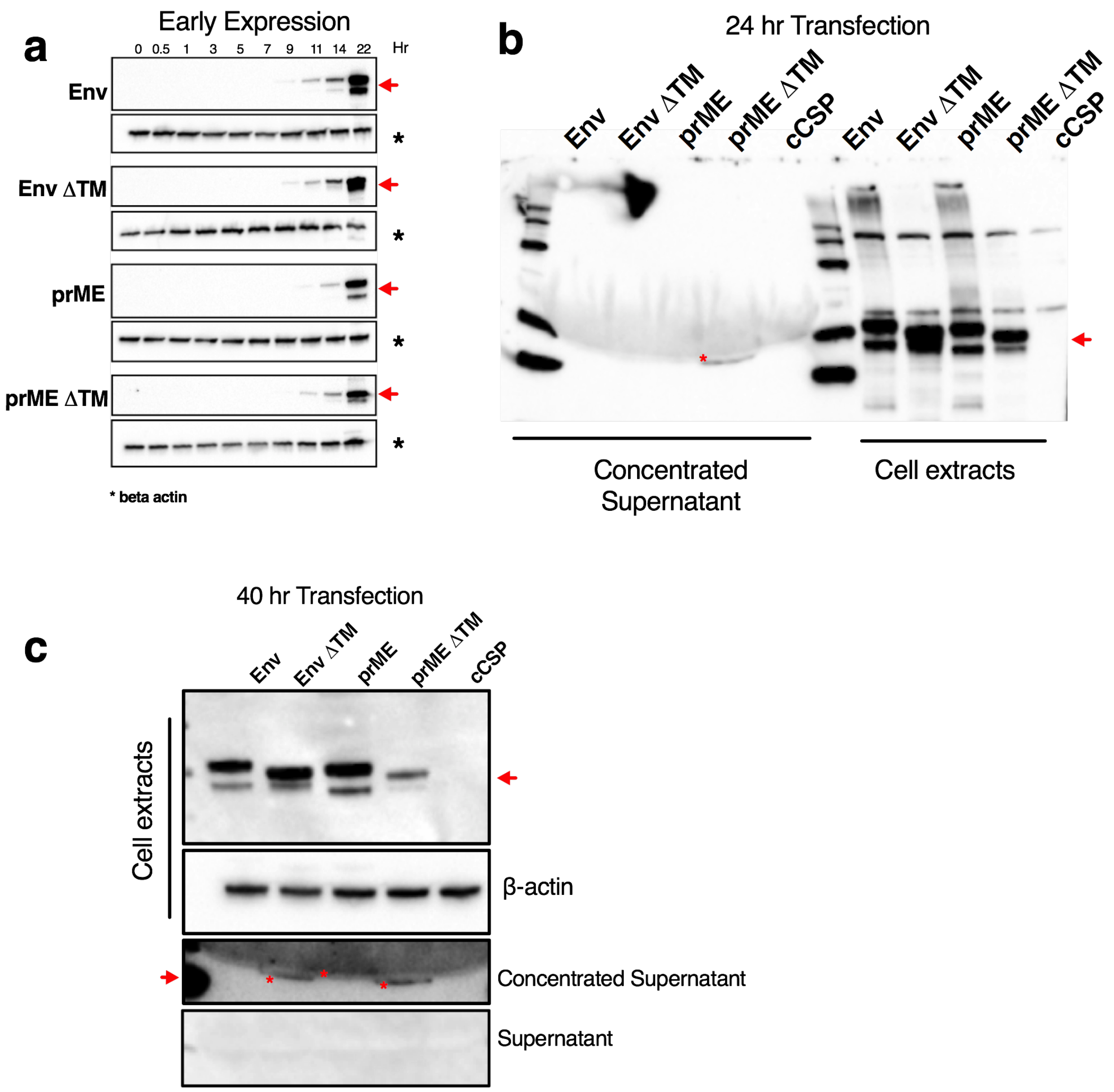

Supplementary Figure 9. (**a**) Kinetics of ZIKV envelope expression in HEK293 cell extracts by western-blot. (**b**) 24 hours and (**c**) 40 hours post-transfection for cell extracts and supernatant. Antigen was detected using an anti-ZIKV monoclonal antibody in both supernatant and cell extracts. Red asterisks and red arrows show ZIKV envelope. Actin was used as a loading control (*). Envelope was detected in concentrated supernatant (from different blot shown in Supplementary Fig 11) but not signal was found in non-concentrated supernatant (15ul per well). Uncropped blots are shown in Supplementary Figure 9.

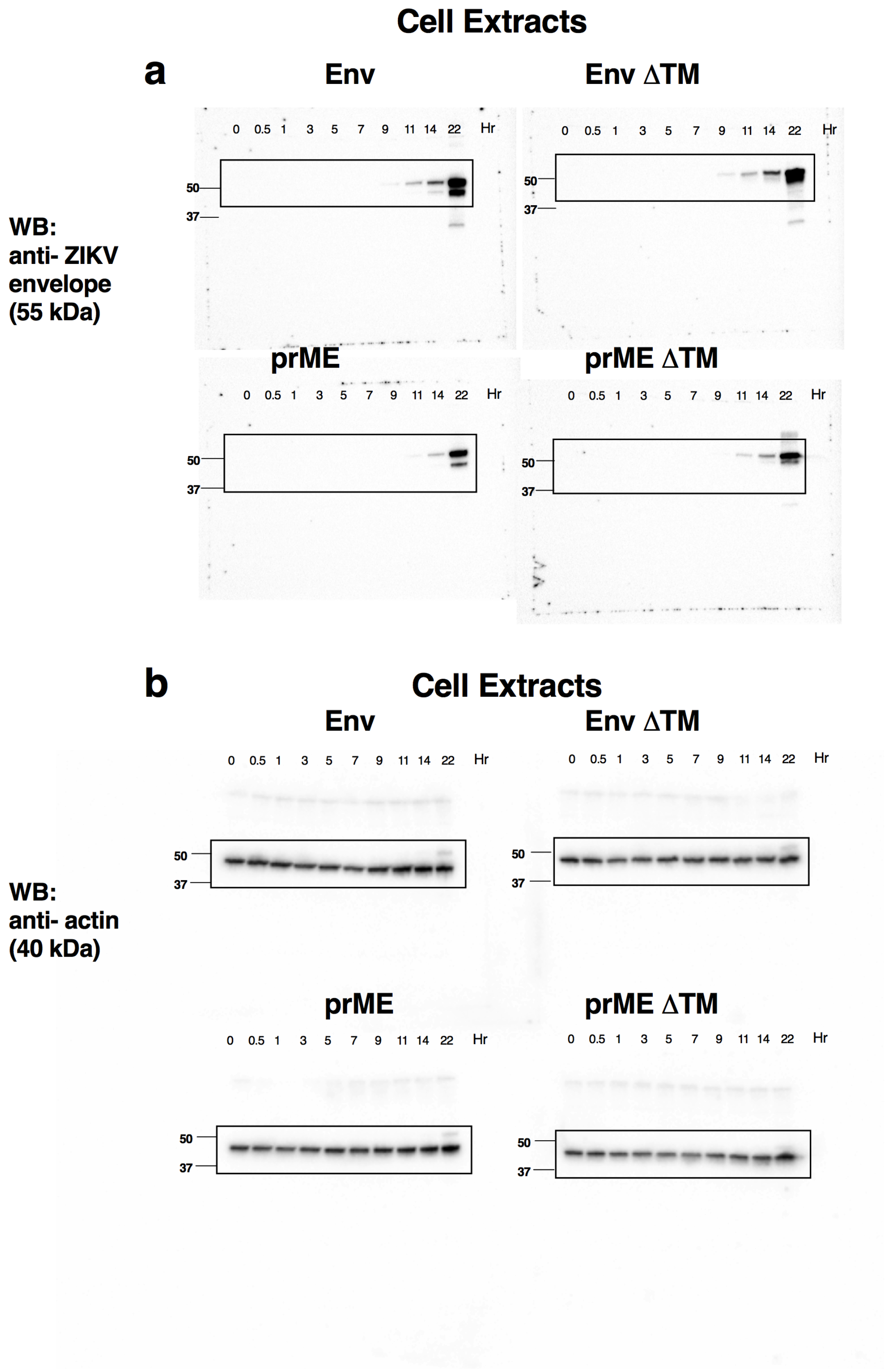

Supplementary Figure 10. Uncropped images from supplementary Figure 8a. (a) ZIKV envelope and (b) actin were detected using the same blot

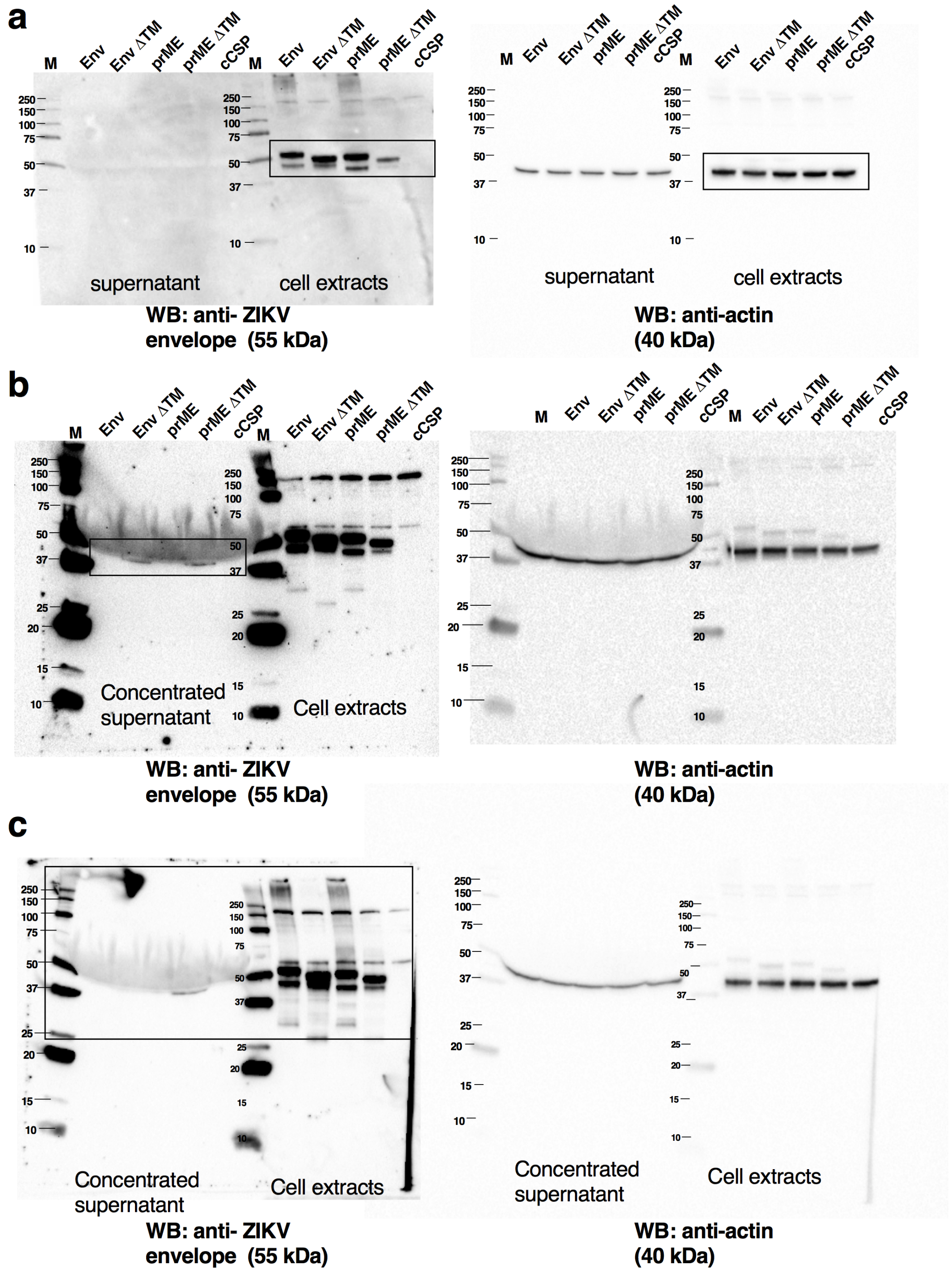

Supplementary Figure 11. Uncropped images from supplementary Figure 8b,c. (**a**) 40 hours post-transfection (left). Actin was detected using the same blot (right). (**b**) Same samples in (a) but using concentrated supernatant instead (left). Actin was detected using the same blot (right). (**c**) 24 hours post-transfection (left), using concentrated supernatant. Actin was detected using the same blot (right).

Microneutralisation (Statistical analysis, multiple comparison between groups)

2 Way ANOVA Multiple Comparisons			
	Significant	Summary	P value
4 weeks:ChAdOx1 Env ΔTM vs. 4 weeks:ChadOx1 prME	No	ns	0.0522
4 weeks:ChAdOx1 Env ΔTM vs. 4 weeks:ChadOx1 prME ΔTM	Yes	**	0.0055
4 weeks:ChAdOx1 Env ΔTM vs. 4 weeks:ChadOx1 Env	No	ns	>0.9999
4 weeks:ChAdOx1 Env ΔTM vs. 16 weeks:ChAdOx1 Env ΔTM	No	ns	>0.9999
4 weeks:ChAdOx1 Env ΔTM vs. 16 weeks:ChadOx1 prME	No	ns	>0.9999
4 weeks:ChAdOx1 Env ΔTM vs. 16 weeks:ChadOx1 prME ΔTM	Yes	****	<0.0001
4 weeks:ChAdOx1 Env ΔTM vs. 16 weeks:ChadOx1 Env	No	ns	>0.9999
4 weeks:ChadOx1 prME vs. 4 weeks:ChadOx1 prME ΔTM	No	ns	>0.9999
4 weeks:ChadOx1 prME vs. 4 weeks:ChadOx1 Env	No	ns	0.0546
4 weeks:ChadOx1 prME vs. 16 weeks:ChAdOx1 Env ΔTM	No	ns	0.0617
4 weeks:ChadOx1 prME vs. 16 weeks:ChadOx1 prME	No	ns	0.3766
4 weeks:ChadOx1 prME vs. 16 weeks:ChadOx1 prME ΔTM	Yes	****	<0.0001
4 weeks:ChadOx1 prME vs. 16 weeks:ChadOx1 Env	No	ns	0.0572
4 weeks:ChadOx1 prME ΔTM vs. 4 weeks:ChadOx1 Env	Yes	**	0.0058
4 weeks:ChadOx1 prME ΔTM vs. 16 weeks:ChAdOx1 Env ΔTM	Yes	**	0.0066
4 weeks:ChadOx1 prME ΔTM vs. 16 weeks:ChadOx1 prME	No	ns	0.0599
4 weeks:ChadOx1 prME ΔTM vs. 16 weeks:ChadOx1 prME ΔTM	Yes	***	0.0003
4 weeks:ChadOx1 prME ΔTM vs. 16 weeks:ChadOx1 Env	Yes	**	0.0061
4 weeks:ChadOx1 Env vs. 16 weeks:ChAdOx1 Env ΔTM	No	ns	>0.9999
4 weeks:ChadOx1 Env vs. 16 weeks:ChadOx1 prME	No	ns	>0.9999
4 weeks:ChadOx1 Env vs. 16 weeks:ChadOx1 prME ΔTM	Yes	****	<0.0001
4 weeks:ChadOx1 Env vs. 16 weeks:ChadOx1 Env	No	ns	>0.9999
16 weeks:ChAdOx1 Env ΔTM vs. 16 weeks:ChadOx1 prME	No	ns	>0.9999
16 weeks:ChAdOx1 Env ΔTM vs. 16 weeks:ChadOx1 prME ΔTM	Yes	****	<0.0001
16 weeks:ChAdOx1 Env ΔTM vs. 16 weeks:ChadOx1 Env	No	ns	>0.9999
16 weeks:ChadOx1 prME vs. 16 weeks:ChadOx1 prME ΔTM	Yes	****	<0.0001
16 weeks:ChadOx1 prME vs. 16 weeks:ChadOx1 Env	No	ns	>0.9999
16 weeks:ChadOx1 prME ΔTM vs. 16 weeks:ChadOx1 Env	Yes	****	<0.0001

Supplementary Table 1. Table shows the statistical analysis (Two-way ANOVA, Multiple comparison between vaccinated groups) with P values.
